# A Comparison of Empirical Correlations of Viscosity and Thermal Conductivity of Water-Ethylene Glycol-Al_2_O_3_ Nanofluids

**DOI:** 10.3390/nano10081487

**Published:** 2020-07-29

**Authors:** Dorota Sawicka, Janusz T. Cieśliński, Slawomir Smolen

**Affiliations:** 1 Faculty of Nature and Engineering, City University of Applied Sciences Bremen, Neustadtswall 30, 28 199 Bremen, Germany; dorota.czerwonka@gmail.com (D.S.); Slawomir.Smolen@hs-bremen.de (S.S.); 2 Faculty of Mechanical Engineering, Gdańsk University of Technology, Narutowicza 11/12, 80233 Gdansk, Poland

**Keywords:** nanofluids, thermophysical properties, correlations

## Abstract

Because of their superb thermal conductivity, nanofluids are seen as new generation of cooling mediums in many engineering applications. It is well established that even a small amount of nanoparticles mixed with a base fluid may result in distinct thermal conductivity enhancement. On the other hand, addition of nanoparticles to the base fluid results in its substantial viscosity increase. Therefore, it is very difficult to evaluate the relative importance of viscosity and thermal conductivity of the nanofluid on convective heat transfer performance. In order to estimate such resultant impact properly, it is necessary to develop reliable correlation equations for predictions of these two thermophysical properties of nanofluids. In this paper, the thermal conductivity and dynamic viscosity of five fluids, i.e., pure water, ethylene glycol (EG) and three mixtures of water and EG with volume ratio of 40:60, 50:50 and 60:40 have been experimentally determined. The aforementioned fluids served as base fluids in nanofluids with Al_2_O_3_ nanoparticles at the concentration of 0.01%, 0.1% and 1% by weight. A set of 20 correlations for prediction of thermal conductivity and dynamic viscosity of base fluids and corresponding nanofluids has been developed. Moreover, present results have been confronted with literature data and predictions made by use of carefully selected recognized literature correlations.

## 1. Introduction

The remarkable thermophysical properties of nanofluids makes them interesting alternatives to common working fluids in many thermal systems, such as solar technologies, refrigeration, combustion engines, cooling of electronic devices, engine radiators and nuclear reactors [1,2,3]. However, the application of nanofluids in engineering requires, among other factors, reliable formulas for the prediction of the thermophysical properties of nanofluids. Unfortunately, existing theoretical models—mostly developed for mixtures (slurries) with milli- and microparticles—fail totally in many cases [4,5]. Knowledge of formulas for the prediction of the thermophysical properties of nanofluids in an analytical form is indispensable for generalisation of the experimental data in the form of Nusselt-type correlations or for numerical modelling of heat transfer processes. Of particular interest are correlations encompassing nanoparticle concentration and temperature jointly [6].

Because of its low freezing point, the application of ethylene glycol in thermal systems is of particular interest in cold regions. However, compared to water, the specific heat of EG is almost two times lower and its viscosity at room temperature is almost four orders of magnitude higher [7]. It seems that a mixture of water and EG as a working fluid is a promising solution in order to improve the specific heat and simultaneously to avoid a significant increase of pumping power. Thus, Nazari et al. [8] examined the CPU performance by using EG/water (30:70 by volume) and EG/water (50:50 by volume) mixtures as base fluids with different nanoparticle concentrations of Al_2_O_3_ and CNT. They observed that EG/water mixtures display better cooling performance than pure water. The final CPU temperature is reduced by ~22% when using CNT in EG/water (30:70 by volume) nanofluid. Recently, Alfaryjat et al. [9] tested EG/water (20:80 by volume) mixture as a base fluid with dispersed CeO_2_, Al_2_O_3_ and ZrO_2_ nanoparticles as a cooling medium in a microchannel heat sink. An increase in the heat transfer coefficient up to 29% was observed compared with the base fluid. Although the operation temperature ranged from 25 °C to 40 °C, the thermophysical properties of the tested nanofluids were determined only at 25 °C.

A limited number of works is devoted to the thermophysical properties of water–EG mixture-based nanofluids. Namburu et al. [10] have developed new viscosity correlations as a function of temperature and volume concentration for silicon dioxide (SiO_2_) and alumina (Al_2_O_3_) nanoparticles in EG/water (60:40 by mass) base fluid. Sahoo et al. [11] have proposed two new correlations expressing viscosity as a function of temperature and Al_2_O_3_ concentration EG/water (60:40 by mass) base fluid for temperatures up to 90 °C. Vajjha and Das [12] have developed a general correlation for the specific heat as a function of particle volumetric concentration, temperature, and the specific heat of both the particle and the base fluid EG/water (60:40 by mass) for mixtures containing aluminium oxide (Al_2_O_3_), zinc oxide (ZnO) and silicon dioxide (SiO_2_) nanoparticles. Vajjha and Das [13] have proposed the thermal conductivity correlation for 60:40% EG/W-based nanofluids containing Al_2_O_3_, ZnO and CuO nanoparticles. Elias et al. [14] have determined the viscosity, thermal conductivity, density and specific heat of Al_2_O_3_ nanoparticles suspended in a radiator coolant (50:50 mixtures of EG/water). Sundar et al. [15] have proposed correlations for the thermal conductivity and viscosity of EG/water mixtures (20:80, 40:60 and 60:40 by mass) with dispersed Al_2_O_3_ nanoparticles. Chiam et al. [16] have developed correlations for the thermal conductivity and dynamic viscosity for Al_2_O_3_ nanoparticles dispersed in water and EG mixtures with ratio of 40:60, 50:50 and 60:40 (by volume) as a function of nanoparticle concentration, temperature and nanoparticle diameter. Recently, Sekrani and Poncet [17] reviewed studies on the thermophysical properties and performances of EG or propylene-glycol (PG)-based nanofluids.

In this paper, a set of new correlations for thermal conductivity and dynamic viscosity of base fluids, i.e., water, EG and water/EG mixtures with volume ratio of 40:60, 50:50 and 60:40, are proposed. Moreover, a set of new correlations for the thermal conductivity and dynamic viscosity of water and water/EG mixtures with ratio of 40:60, 50:50 and 60:40 as base fluids for nanofluids with dispersed Al_2_O_3_ nanoparticles is developed. The nanofluids were tested at the nanoparticle mass concentrations of 0.01%, 0.1% and 1%. The proposed new correlations are confronted with some relevant reference data and predictions made by use of selected published correlations for nanofluids with dispersed Al_2_O_3_ nanoparticles.

## 2. Tested Fluids

In this study, alumina (Al_2_O_3_) nanoparticles were used while distilled, deionized water, ethylene glycol (EG) and three mixtures of water and EG with volume ratio of 40:60, 50:50 and 60:40 were applied as the base fluids. Water and pure EG were provided by PanReac AppliChem (AppliChem GmbH, ITW Reagents, Darmstadt, Germany), and the water/EG mixtures were prepared at the laboratory. Nanofluids with different concentrations were fabricated for the experiments.

Nanoparticles of the required amount and the base liquid were mixed together. Ultrasonic vibration was used for 4 h in order to stabilise the dispersion of the nanoparticles. Alumina nanoparticles were tested at the concentrations of 0.01%, 0.1% and 1% by weight. Alumina (Al_2_O_3_) nanoparticles of spherical form have diameters from 5 nm to 250 nm; their mean diameter was estimated to be 47 nm according to the manufacturer (Sigma-Aldrich Co, Merck KGaA, Darmstadt, Germany).

### 2.1. Viscosity

For determination of the viscosity of the tested liquids the capillary viscometer Rheotest LK 2.2 of Rheotest Medingen GmbH (Ottendorf-Okrilla, Germany) was used. To obtain high accuracy of the measurement a special capillary measuring the viscosity from 1 to 16 mPas was implemented. This viscometer measures the dynamic viscosity predominant of Newtonian fluids within the viscosity range of 1 to 10,000 mPas (for different capillaries) within the temperature range from 10 °C to 80 °C and accuracy below ±2%. Measurement time equals 35 s. The analysis was performed under the constant temperature conditions. For this aim, the thermostat Lauda Alpha A6 of the Lauda-Brinkmann (Lauda DR. R. Wobser GmbH & Co. KG, Lauda-Königshofen, Germany) was coupled to the viscometer. In order to avoid impact of the potential nanoparticle agglomeration, the viscosity of fresh prepared nanofluid was measured. Details of the measurement set-up and procedure are described in [18,19].

#### 2.1.1. Base Fluids

It is well-known fact that the viscosity of the fluids is a strong function of the temperature [11,14]. Therefore, an exponential function was selected in order to correlate the present results. Regression analysis using the least squares method was applied to correlate experimentally obtained data for five tested base fluids. The correlations used to predict the dynamic viscosity of the base fluids are summarised in Table 1.

**Figure 1:**
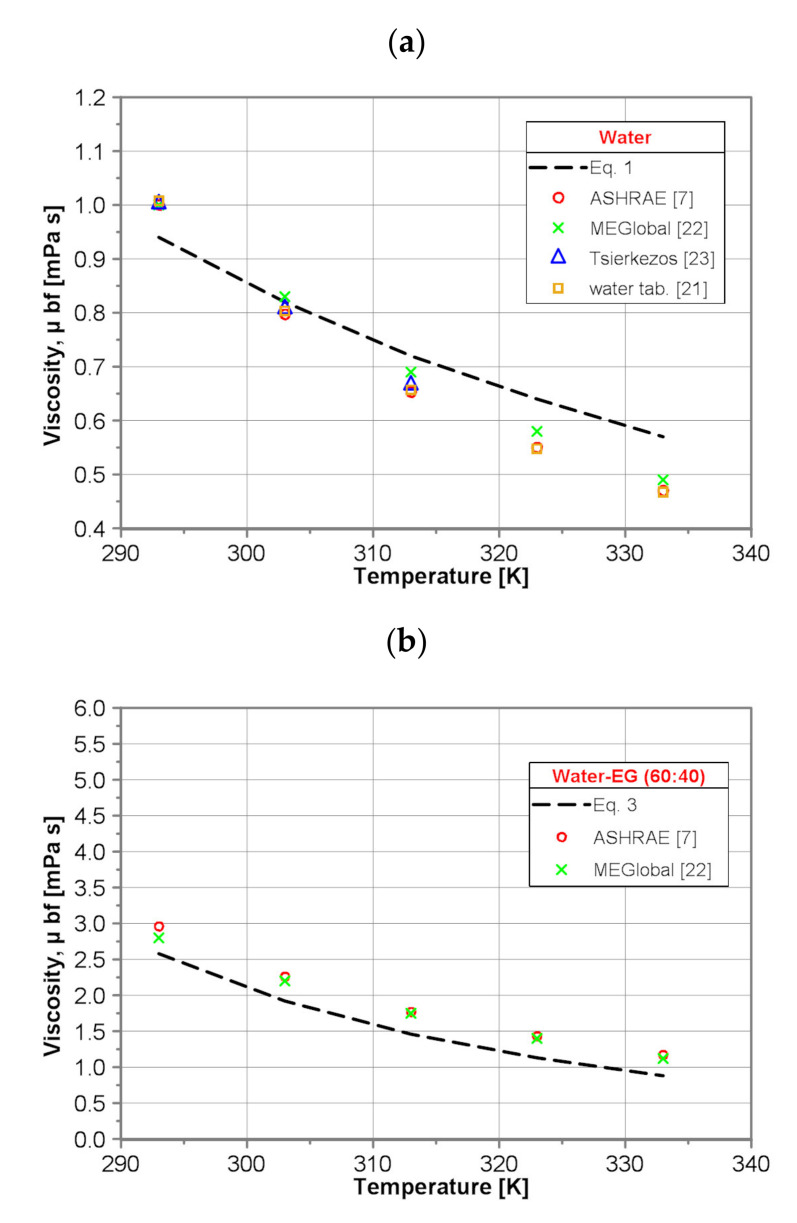

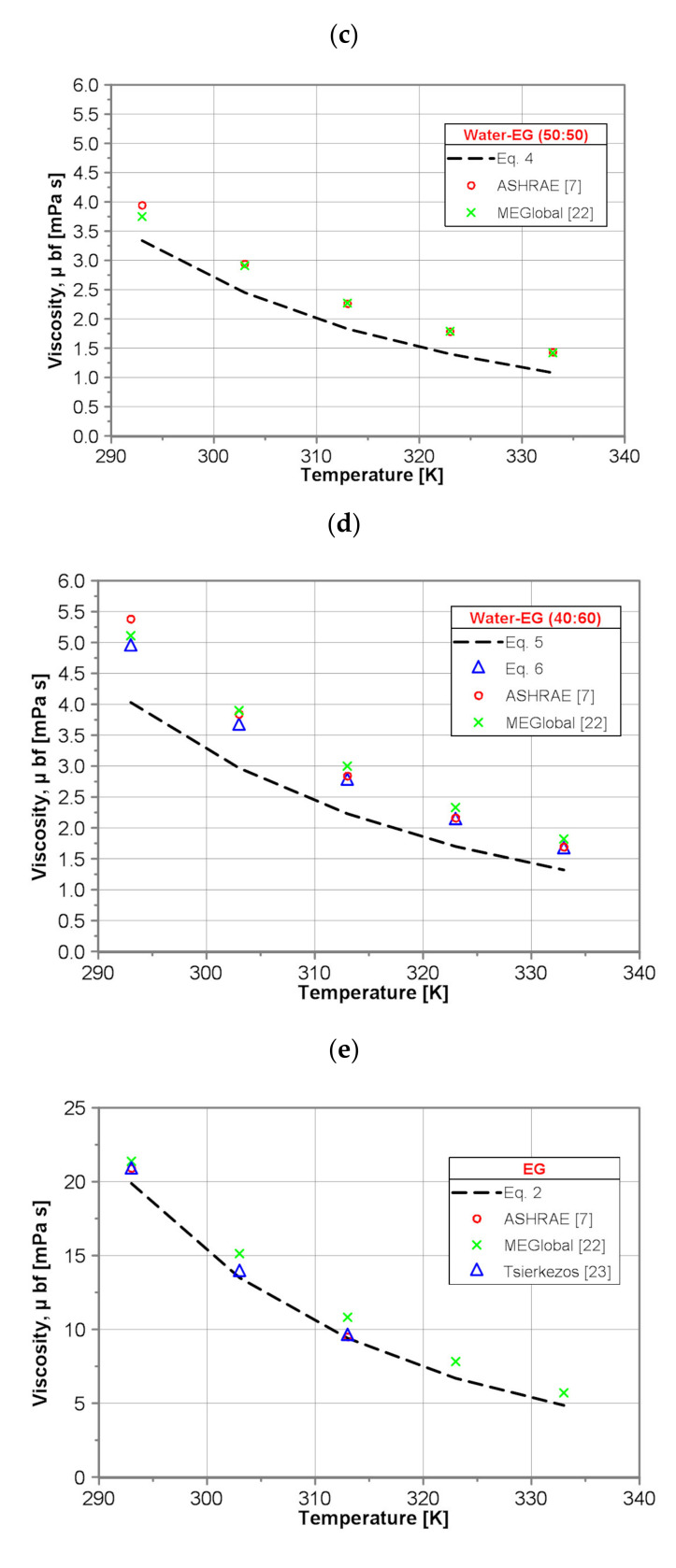
Comparison of the base fluids dynamic viscosity; (**a**) water, (**b**) water/EG(60:40), (**c**) water/EG(50:50), (**d**) water/EG(40:60) and (**e**) EG.

In order to check the credibility of the present results, a comparison of the calculated values of the dynamic viscosity of the base fluids by use of the developed correlations (Equations (1–5)) with the data published in the literature for the temperature range of 293 to 333 K has been conducted. As seen in Figure 1, an at least qualitative agreement with the published data [7,20,21,22,23] has been achieved.

It was found that for pure water the values of dynamic viscosity predicted by use of developed Equation (1) are lower than those presented in the literature within the whole tested temperature range. For lower temperature, Equation (1) underpredicts by ~6%, while for higher temperature, Equation (1) overpredicts the data presented in the literature [7,20,21,22,23] by ~17%.

The agreement between the values predicted by use of developed Equation (2) and those presented in the literature for pure EG is much better than for pure water, although Equation (2) underpredicts the dynamic viscosity of EG compared to the published data [7,22,23] within the whole tested temperature range. The maximum difference between the values of dynamic viscosity presented in the literature, i.e., in [7,22,23] equals 14%. The maximum difference between the values of dynamic viscosity predicted by use of Equation (2) and presented in the literature [23] equals 17%.

For all three examined water/EG mixtures, i.e., (60:40), (50:50) and (40:60), the corresponding correlations, i.e., Equation (3), Equation (4) and Equation (5), underpredict the dynamic viscosity of the water/EG mixtures compared to published data [7,20,22] within the whole tested temperature range. For water/EG (60:40), water/EG (50:50) and water/EG (40:60) mixtures, the maximum difference (for the highest temperature) between the values of dynamic viscosity presented in the literature, i.e., in [7,22], equals 33%, 31% and 38%, respectively. It is necessary to stress the reasonable agreement of the predictions obtained by the developed Equation (5) for water/EG (40:60) mixture with results obtained by use of Vajjha and Das correlation (Equation (6)) based on the data provided by ASHRAE Handbook [7]. The difference is almost constant within the whole temperature range and does not exceed 25%.

**Figure 2:**
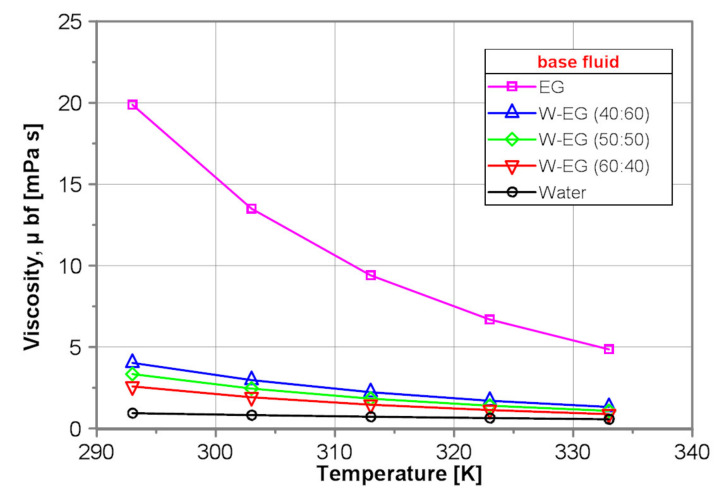
Dynamic viscosity of the tested base fluids.

Figure 2 shows a comparison of the dynamic viscosity of the tested base fluids obtained by use of the proposed correlation equations presented in Table 1. As seen in Figure 2, the dynamic viscosity of EG is over 20 times higher for 293 K and almost order of magnitude higher for 333 K than that of water. Dynamic viscosity of all tested base fluids decreases with temperature, but the rate of the decrease is for EG the highest. Dynamic viscosity of the water–EG mixtures decreases distinctly with increasing water content.

#### 2.1.2. Nanofluids

The present data of the measured viscosity of the tested nanofluids were correlated by use of the functions in the form that was originally proposed by Yiamsawas et al. [24]:

(7) $$\mu_{nf}=A\varphi^{B}t^{C}\mu_{bf}^{D}\;\;t[{}^{\circ}C],$$

The developed correlations are presented in Table 2.

In order to check the reliability of the present results, a comparison of the calculated values of the dynamic viscosity of the tested nanofluids with the predictions made by the correlations published in the literature has been conducted.

The Sahoo et al. [11] correlation of dynamic viscosity of EG/water (60:40 by mass)/Al_2_O_3_ nanofluid for temperature range 273 K–363 K is as follows.

(13) $$\mu_{nf}=2.392\cdot 10^{-4}\cdot e^{\left(\frac{2903}{T}+0.1265\varphi_{v}\right)}$$

The Sundar et al. [15] correlation for dynamic viscosity of EG/water mixtures with dispersed Al_2_O_3_ nanoparticles is as follows.

For mixture EG/water (40:60 by mass)

(14) $$\mu_{nf}=0.9299\cdot \mu_{bf}e^{\left(67.43\varphi_{v}\right)}$$

For mixture EG/water (60:40 by mass)

(15) $$\mu_{nf}=1.1216\cdot \mu_{bf}e^{\left(77.56\varphi_{v}\right)}$$

The Chiam et al. [16] correlation for dynamic viscosity of water/EG mixtures with ratio (BR) of 40:60, 50:50 and 60:40 by volume with seeded Al_2_O_3_ nanoparticles is as follows.

(16) $$\mu_{nf}=\mu_{bf}\left[{\left(1+\frac{\varphi_{v}}{100}\right)}^{32}{\left(\frac{T}{70}\right)}^{-0.001}{\left(0.1+BR\right)}^{0.08}\right]$$

Khanafer and Vafai [4] proposed the following correlation for dynamic viscosity of water/Al_2_O_3_ nanofluids.

(17) $$\mu_{nf}=-0.4491+\frac{28.837}{t}+0.574\varphi_{v}-0.1634\varphi_{v}^{2}+23.053\frac{\varphi_{v}^{2}}{t^{2}}+0.0132\varphi_{v}^{3}\;-2354.735\frac{\varphi_{v}}{t^{3}}+23.498\frac{\varphi_{v}^{2}}{d_{p}^{2}}-3.0185\frac{\varphi_{v}^{3}}{d_{p}^{2}}$$

Building on their own measurements, Pastoriza-Gallego et al. [25] proposed the following correlation for dynamic viscosity of EG-Al_2_O_3_ and water-Al_2_O_3_ nanofluids,

(18) $$\mu_{nf}=e^{\left(A+\frac{B}{T-T_{o}}\right)}$$

where adjustable parameters A, B and *T*_o_ are given in Table 3.

**Figure 3:**
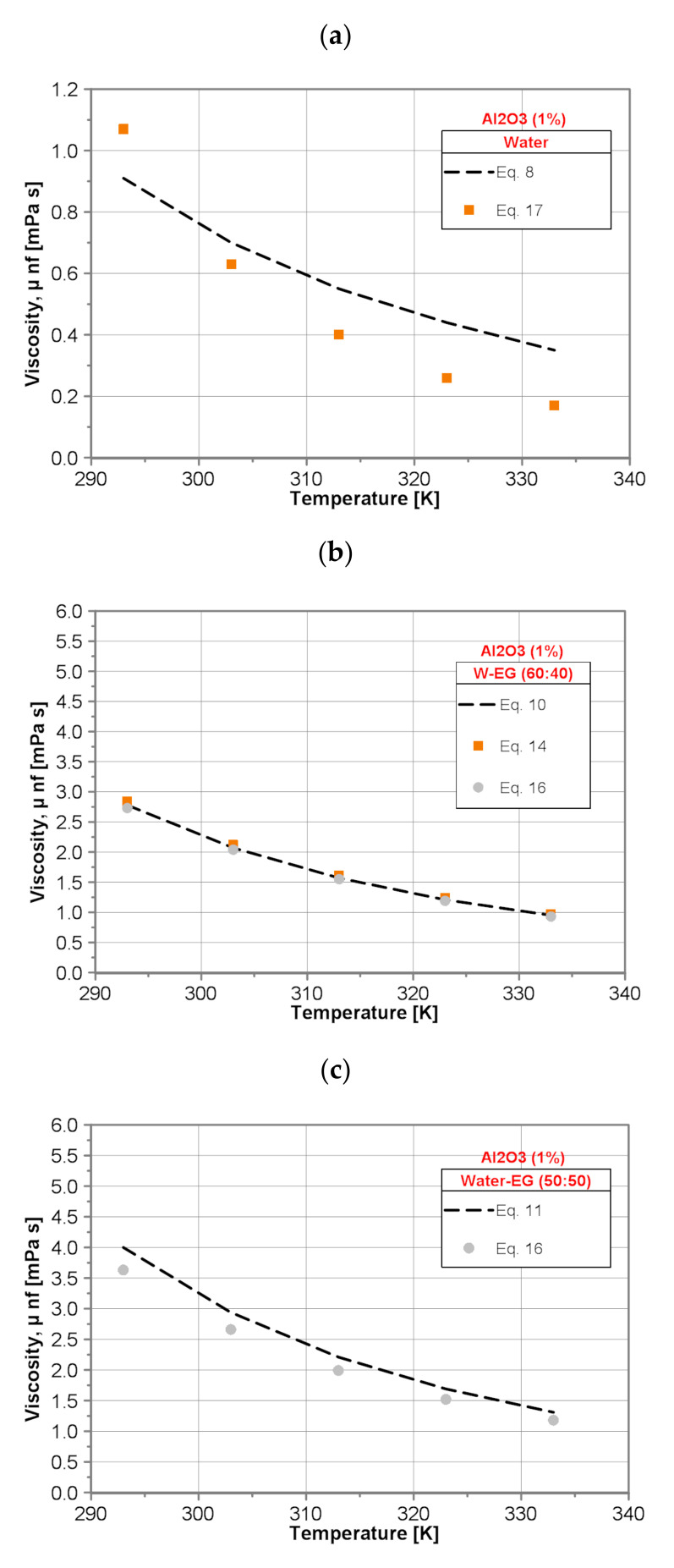

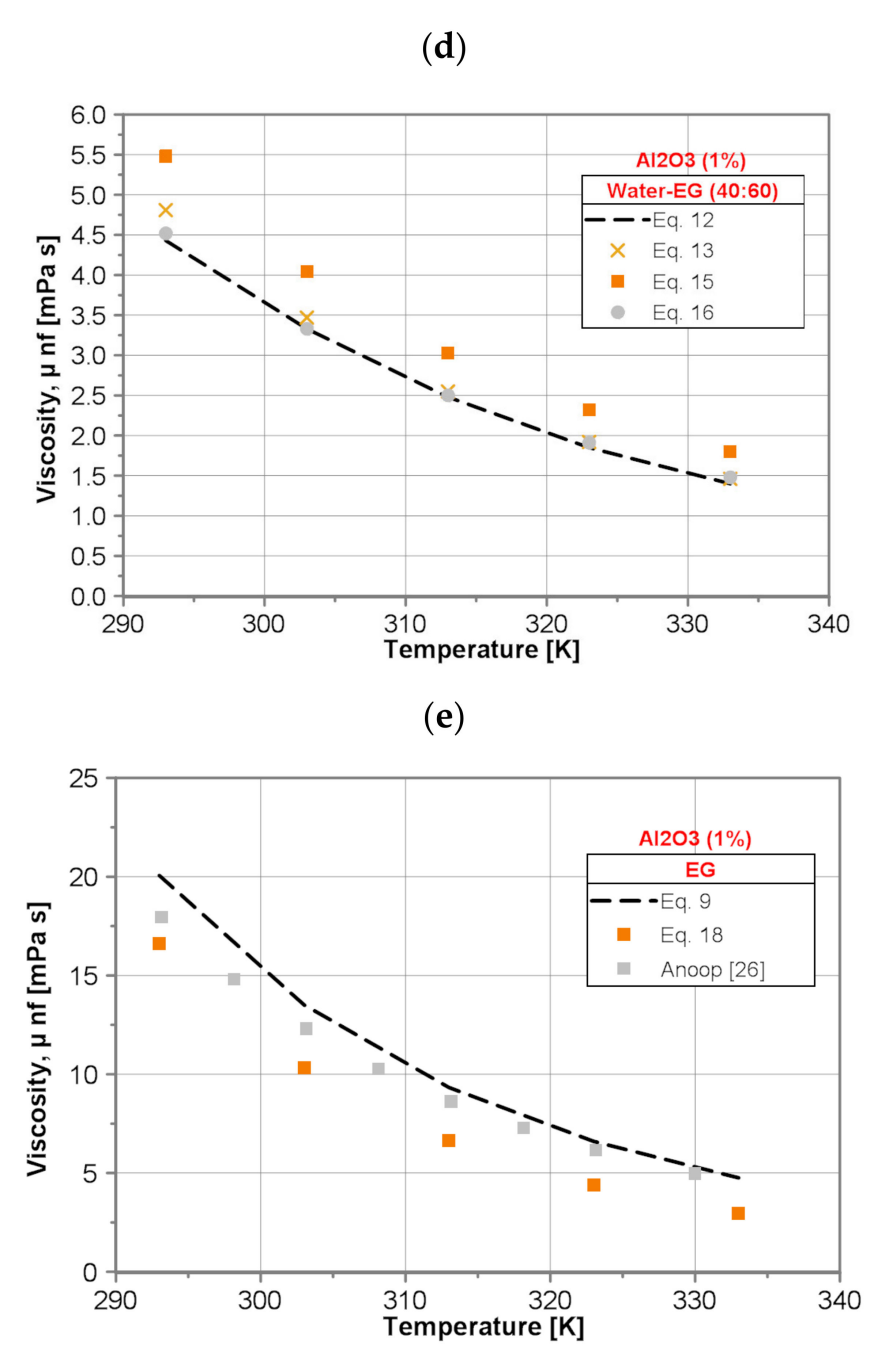
Comparison of dynamic viscosity of the tested nanofluids with 1% nanoparticle mass concentration: (**a**) water/Al_2_O_3_, (**b**) water/EG (60:40)/Al_2_O_3_, (**c**) water/EG(50:50)/Al_2_O_3_, (**d**) water/EG (40:60)/Al_2_O_3_ and (**e**) EG-Al_2_O_3._

As an example, Figure 3 shows the comparison of the dynamic viscosity values of water/EG/Al_2_O_3_ nanofluids for a nanoparticle mass concentration of 1% calculated with the present developed correlations (Equations (8–12)) with predictions made by use of published correlations. The values of dynamic viscosity of water/Al_2_O_3_ (1 wt%) nanofluid predicted by use of developed Equation (8) are smaller (−17.5%) and higher (+51%) than those calculated by Khanafer and Vafai correlation (Equation (17)) for lower and higher temperature, respectively. However, it is necessary to remember that the general correlation of Khanafer and Vafai (Equation (17)) has been developed for distinctly higher nanoparticle concentration (1% ≤ φ ≤ 9%) than in the present study (0.003% ≤ φ ≤ 0.3%). Equation (9) overpredicts the data calculated by use of the Pastoriza-Gallego et al. correlation (Equation (18)) for EG-Al_2_O_3_ (1 wt%) nanofluid within the whole range of temperature tested and the mean deviation is about 22%. Equation (9) overpredicts the experimental data of Anoop et al. [26] for lower temperature by about 10% and underpredicts for higher temperature by ~4%.

Excellent agreement has been observed for water/EG (60:40)/Al_2_O_3_(1 wt%) nanofluid between predictions made by use of developed Equation (10) and correlations proposed by Sundar et al. (Equation (14)) and Chiam et al. (Equation (16)). The differences between the predictions obtained by present (Equation (10)) and literature correlations (Equations (14) and (16)) are almost constant within the whole temperature range and do not exceed +2.5% and −2%, respectively.

For the water/EG (50:50)/Al_2_O_3_(1 wt%) nanofluid, present developed Equation (11) overpredicts the data calculated by use of the Chiam et al. correlation (Equation (16)) within the whole range of temperature tested and the mean deviation is about 10.5%.

For the water/EG (40:60)/Al_2_O_3_(1 wt%) nanofluid, the results obtained by present developed Equation (12) display excellent agreement with the predictions made by use of Sahoo et al. correlation (Equation (13)) and Chiam et al. correlation (Equation (16)). The difference between the predictions obtained by present (Equation (12)) and literature correlation (Equation (13)) does not exceed 4%, except the value for the lowest temperature (8.5%). The difference between the predictions obtained by present (Equation (12)) and literature correlation (Equation (16)) does not exceed 5%. Much higher discrepancy is observed between the predictions obtained by present (Equation (12)) and literature correlation (Equation (15)). Equation (12) underpredicts the data calculated by use of the Sundar et al. correlation (Equation (15)) within the whole range of temperature tested and the mean deviation is about 24%.

**Figure 4:**
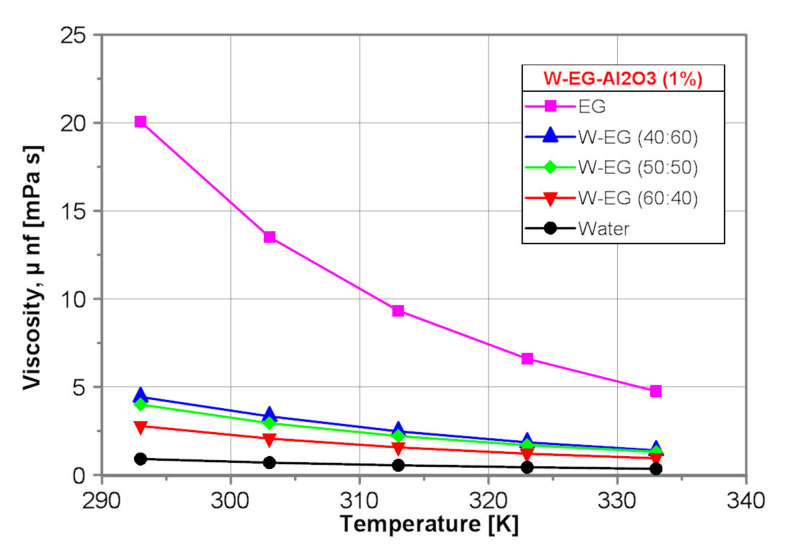
Dynamic viscosity of the tested nanofluids.

Figure 4 shows a comparison of the dynamic viscosity of the tested nanofluids with nanoparticle mass concentration of 1% obtained by use of the proposed correlation equations presented in Table 2.

Similarly to the base fluids, the dynamic viscosity of the EG-Al_2_O_3_ (1%) nanofluid is the highest one. Moreover, the dynamic viscosity of all the tested nanofluids decreases with water content and temperature increase.

**Figure 5:**
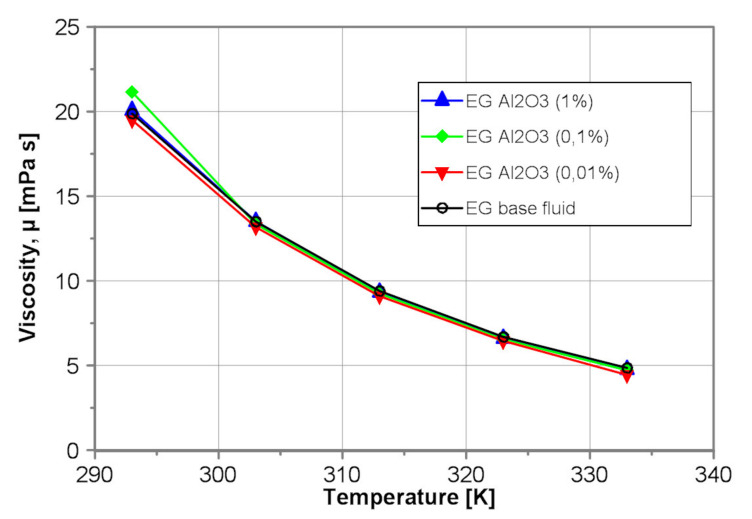
Dynamic viscosity of the tested EG-Al_2_O_3_ nanofluids.

Figure 5, in turn, illustrates the exemplarily influence of the nanoparticle concentration on the dynamic viscosity of the tested EG-Al_2_O_3_ nanofluids. As results from Figure 5, the impact of alumina nanoparticles on dynamic viscosity of EG-Al_2_O_3_ nanofluids within mass concentration from 0.01% to 1% and temperature range 293K ≤ T ≤ 333K is negligible. The discrepancy between the data is within the measurement error.

### 2.2. Thermal Conductivity

In the present study, the thermal conductivity of the tested liquids was measured by use of KD2 Thermal Properties Analyser of the Decagon Devices Inc. (Pullman, WA, USA). With the implemented sensor the measurement was possible for the thermal conductivity range from 0.02 to 2.00 W/(mK) and the temperature range from −20 ℃ to 60 °C. In this work, the thermal conductivity was determined for the temperature range of +20 to +40 °C. Maximum expected uncertainty amounts: ±5%. For the measurement amount of 20 mL of the prepared nanofluid was put in the laboratory cell and placed in the bath of given temperature. The sensor was immersed in the middle of the sample without any contact with the cell walls. The measurement time for every temperature amounted 2 min. In order to avoid impact of potential agglomeration the thermal conductivity of fresh prepared nanofluid was measured (according to the work in [6] many abnormal thermal conductivity data in nanofluids result from unstable nanofluids). The details of the measurement set-up and procedure are described in [18,19].

#### 2.2.1. Base Fluids

Thermal conductivity of the pure fluids weakly depends on the temperature [20]. The correlations used to evaluate the thermal conductivity of the base fluids are developed by assuming that thermal conductivity of the base fluids increases linearly with temperature. Regression analysis, using the least squares method, was applied to correlate experimentally obtained data for five tested base fluids.

The correlations are summarized in Table 4.

**Figure 6:**
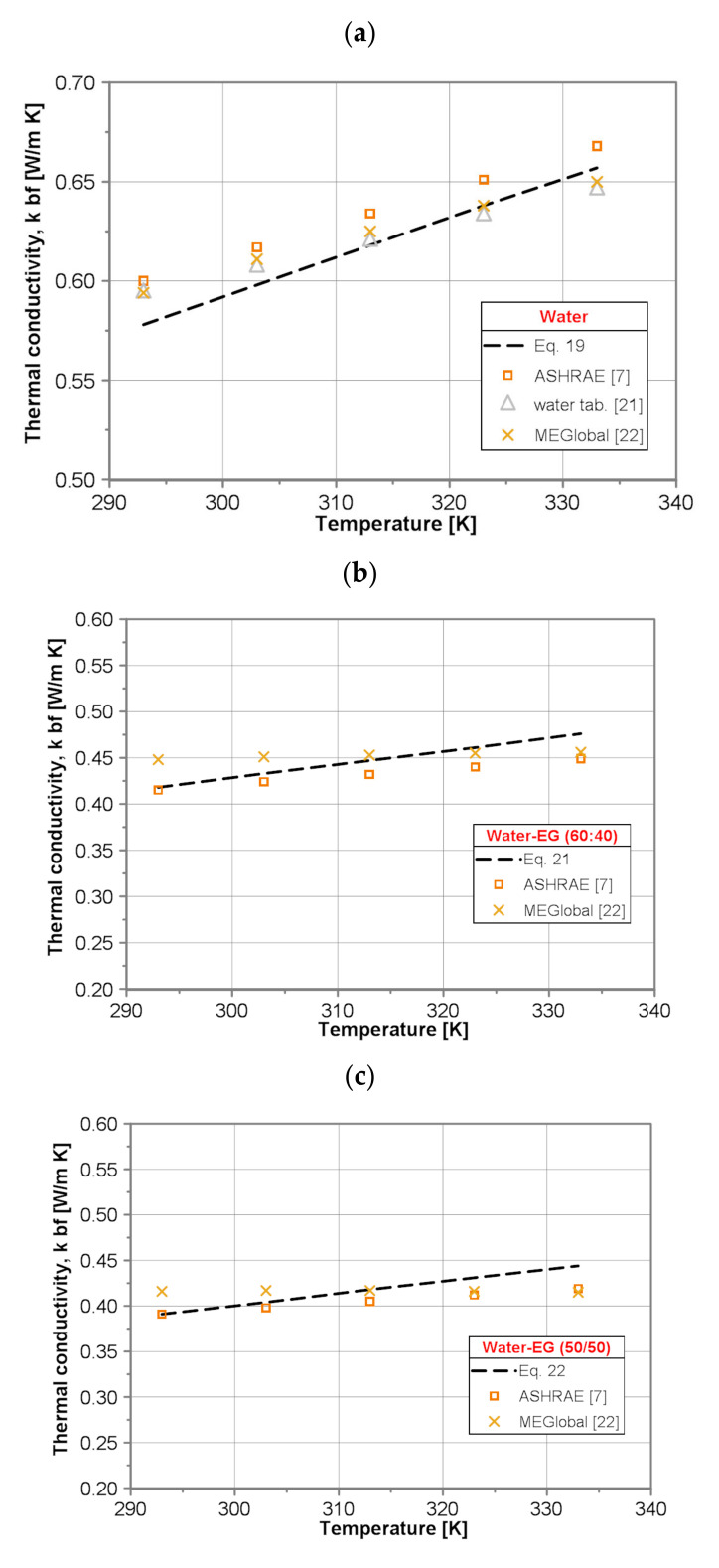

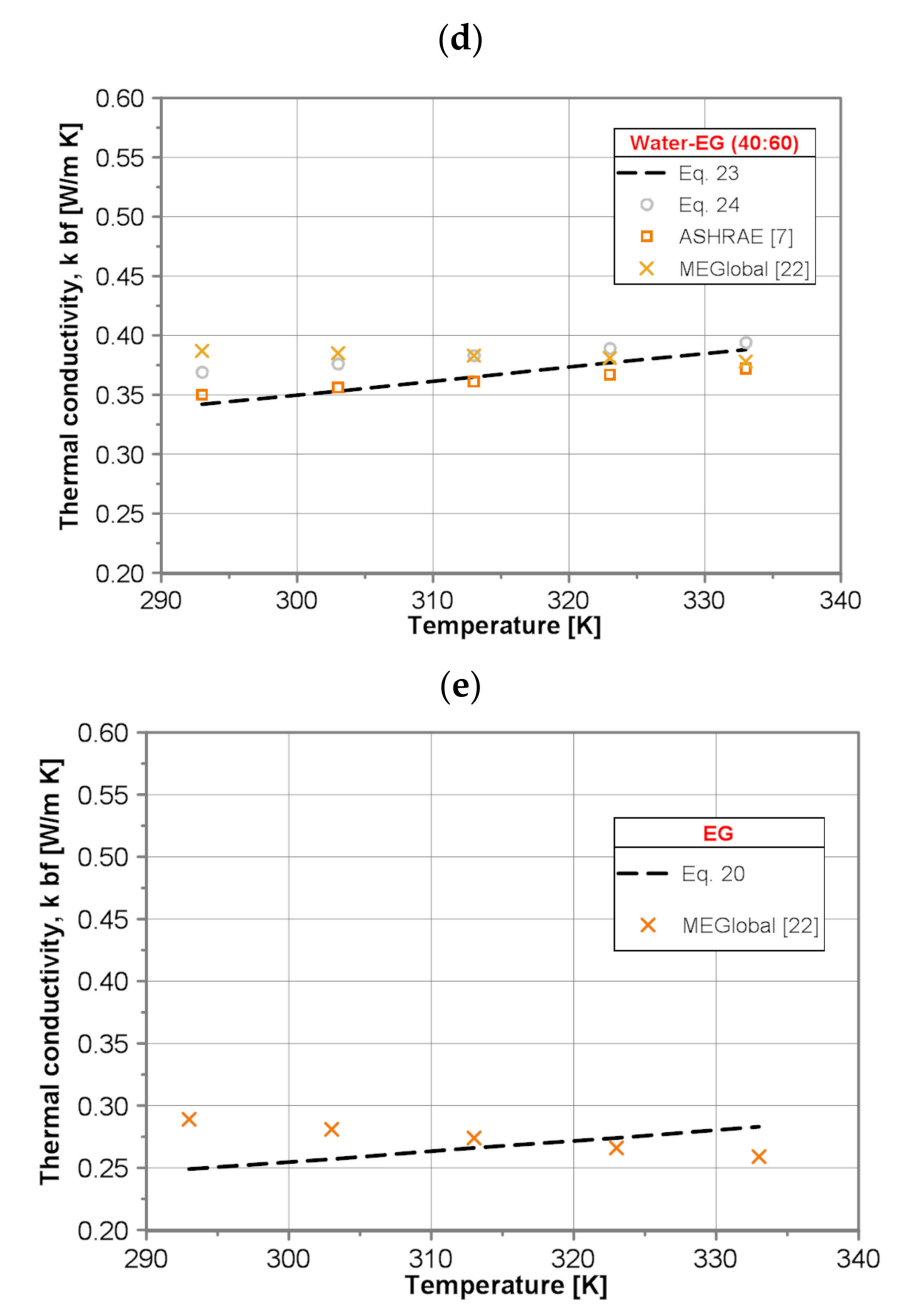
Comparison of the base fluids thermal conductivity: (**a**) water, (**b**) water/EG(60:40), (**c**) water/EG(50:50), (**d**) water/EG(40:60) and (**e**) EG.

In order to check the credibility of the present results, a comparison of the thermal conductivity of the base fluids calculated by the developed correlations (Equations (19–23)) with the data published in the literature for the temperature range of 293 to 333 K has been conducted (see Figure 6).

It was found that for pure water the values of thermal conductivity predicted by use of developed Equation (19) display reasonable agreement with the literature data [7,21,22] within the whole temperature range. The maximum discrepancy reads ±3.5% and is comparable with the deviation between literature data (±3.4%), as well as is within maximum expected measurement uncertainty (±5%).

For pure EG the values predicted by use of developed Equation (20) and those presented in the literature [22] deviate from −16% for lower temperature to +9% for higher temperature.

For all three water/EG mixtures, i.e., (60:40), (50:50) and (40:60), corresponding to Equation (21), Equations (22) and (23) underpredict the thermal conductivity of the water/EG mixtures compared to the published data [7,20,21,22] for lower temperature and overpredict for higher temperature. The maximum discrepancy occurs for data published in [22] and it deviates from −7% to +4 for water/EG (60:40) mixture, from −6% to +6% for water/EG (50:50) mixture and from −13% to +3% for water/EG (40:60) mixture. The Vajjha and Das correlation (Equation (24)) is based on the data provided for water/EG (40:60) mixture in [7], and it is seen in Figure 3 that there is a difference between calculated values and data taken directly from the tables. Correlation (Equation (24)) gives higher values of ~6% within the whole tested temperature range.

**Figure 7:**
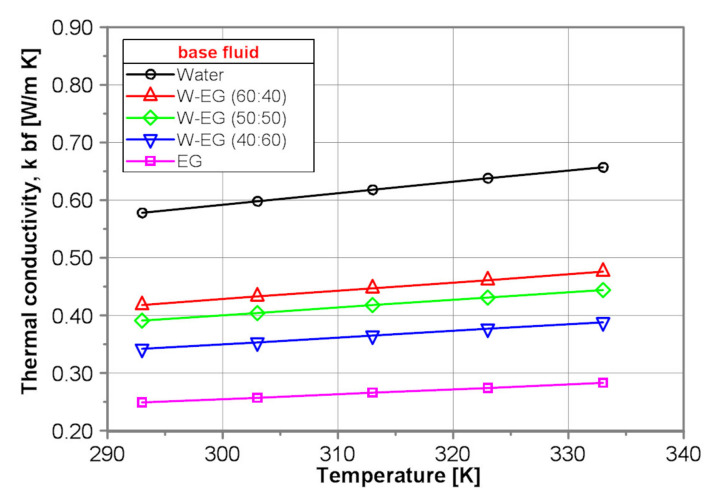
Thermal conductivity of the tested base fluids.

Figure 7 shows a comparison of the thermal conductivity of the tested base fluids obtained by use of the proposed correlation equations presented in Table 4. As it is seen in Figure 7, the thermal conductivity of water is, independent of temperature, over 2 times higher than that of EG. The thermal conductivity of all tested base fluids increases with temperature by ~13%. Thermal conductivity of the water–EG mixtures decreases distinctly with water content decrease.

#### 2.2.2. Nanofluids

The present thermal conductivity data of the tested nanofluids were correlated in the form that was originally proposed by Patel et al. [27]:

(25) $$k_{nf}=k_{bf}(1+A{\left(\frac{k_{p}}{k_{bf}}\right)}^{B}\varphi_{v}^{C}{\left(t/20\right)}^{D}\;{\left(100/d_{p}\right)}^{E}){\;}_{}$$

A multidimensional regression analysis using the least squares method was applied to establish parameters A–E. The developed correlations are presented in Table 5.

In order to check the reliability of the present developed correlations, a comparison of the calculated values of the thermal conductivity of the tested nanofluids with the predictions made by correlations published in the literature has been conducted.

The Sundar et al. [15] correlation for thermal conductivity of EG/water mixtures with dispersed Al_2_O_3_ nanoparticles is as follows.

For mixture EG/water (40:60 by mass)

(31) $$k_{nf}=k_{bf}\left(1.0806+10.164\varphi_{v}\right)$$

For mixture EG/water (60:40 by mass)

(32) $$k_{nf}=k_{bf}\left(1.0618+10.448\varphi_{v}\right)$$

The Chiam et al. [16] correlation for thermal conductivity of water/EG mixtures with ratio (BR) of 40:60, 50:50 and 60:40 by volume with seeded Al_2_O_3_ nanoparticles is as follows.

(33) $$k_{nf}=k_{bf}\left[0.9683{\left(1+\frac{\varphi_{v}}{100}\right)}^{11.13}{\left(1+\frac{T}{70}\right)}^{0.1676}{\left(0.1+BR\right)}^{0.0011}{\left(\frac{d_{p}}{36}\right)}^{0.0572}\right]$$

Khanafer and Vafai [4] proposed the following correlation for water/Al_2_O_3_ nanofluids.

(34) $$k_{nf}=k_{bf}\left(0.9843+0.398\varphi_{v}^{0.7383}{\left(\frac{1}{d_{p}}\right)}^{0.2246}{\left(\frac{\mu_{nf}}{\mu_{bf}}\right)}^{0.0235}-3.9517\frac{\varphi_{v}}{t}+34.034\frac{\varphi_{v}^{2}}{t^{3}}\;+32.509\frac{\varphi_{v}}{t^{2}}\right)$$

Corcione [28] developed a correlation for oxide and metal nanoparticles suspended in water or EG-based nanofluids,

(35) $$k_{nf}=k_{bf}\left(1+4.4Re^{0.4}Pr^{0.66}{\left(\frac{T}{T_{fr}}\right)}^{10}{\left(\frac{k_{p}}{k_{f}}\right)}^{0.03}\varphi_{v}^{0.66}\right)$$

where $Re=\frac{2\rho_{bf}k_{B}T}{\pi \mu_{bf}^{2}d_{p}}$ and k_B_ = 1.3807 · 10^−23^ J/K (Boltzmann constant). Freezing point temperature T_fr_ of the base fluid was determined based on the work in [7].

Hassani et al. [29] proposed a general correlation for various base fluids, metal and oxide nanoparticles in the form

(36) knf=kbf1.04+φv1.11kpkbf0.33Pr−1.71Pr−1.7−262kpkb
f
0.33+135drefdp0.23νbfdpuBr0.82cpT−1   uBr2−0.1TBT−7 

where $u_{Br}={\left(\frac{2k_{B}T}{\pi \varrho_{p}d_{p}^{3}}\right)}^{0.5}$ is the Brownian velocity.

The assumed properties of alumina (Al_2_O_3_) nanoparticles are k_p_ = 35 W/(mK) and ρ_p_ = 3600 kg/m^3^—taken from in [4,30], respectively. The boiling point temperature T_B_ of the base fluid was taken from in [7]. The d_ref_ is the molecular diameter of hydrogen, and is equal to 2.9 Å.

**Figure 8:**
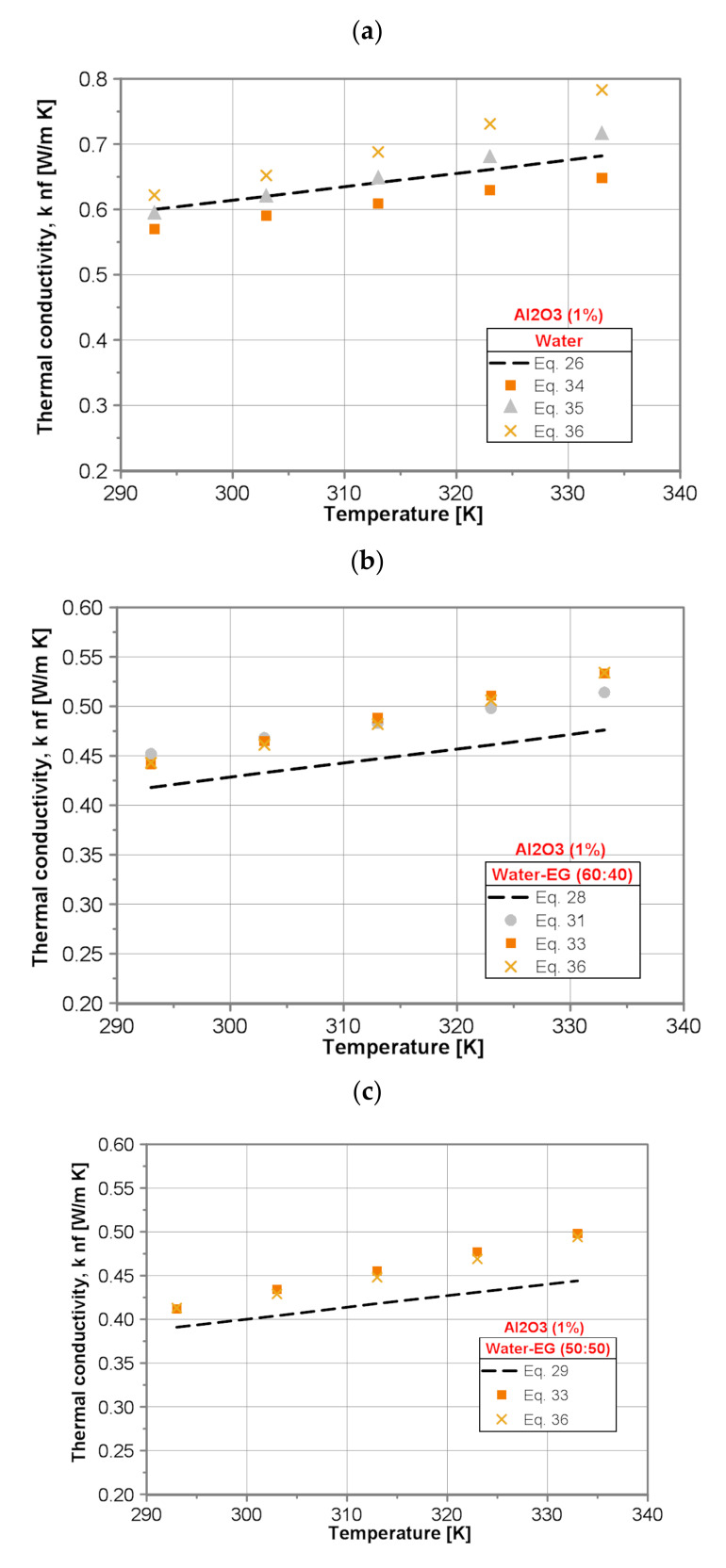

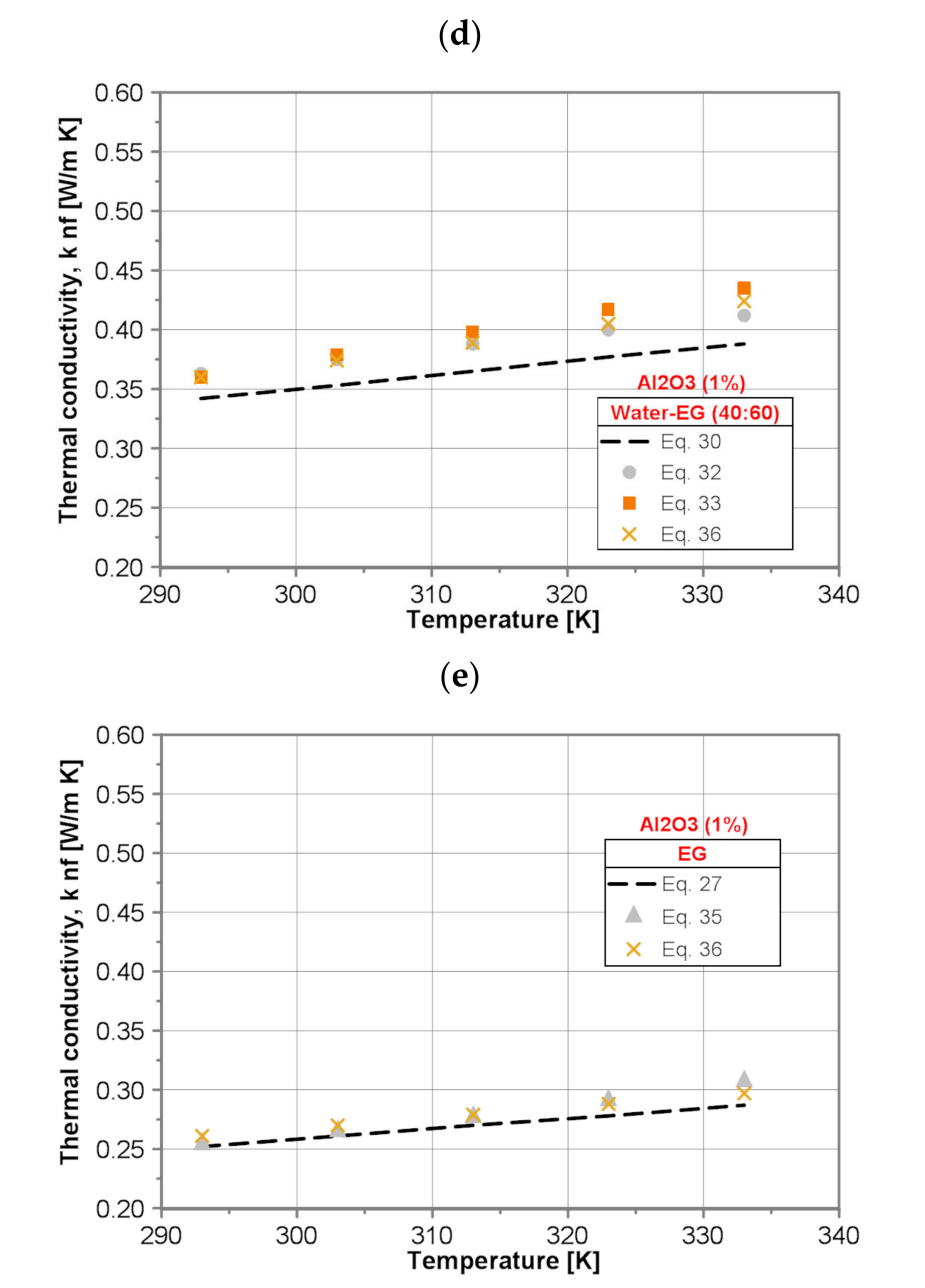
Comparison of thermal conductivity of the tested nanofluids with 1% nanoparticle mass concentration: (**a**) water/Al_2_O_3_, (**b**) water/EG (60:40)/Al_2_O_3_, (**c**) water/EG (50:50)/Al_2_O_3_, (**d**) water/EG (40:60)/Al_2_O_3_ and (**e**) EG-Al_2_O_3._

Figure 8 shows exemplarily the comparison of the measured values of thermal conductivity of water/ Al_2_O_3_ nanofluids for nanoparticle concentration of 1% with literature data.

It was found that for the water/Al_2_O_3_ (1 wt%) nanofluid the values of thermal conductivity predicted by use of developed Equation (26) display reasonable agreement with the predictions made by use of advanced Corcione correlation (Equation (35)). The maximum deviation ranges from +1.3% for lower temperature to −4.7% for higher temperature. The Hassani et al. correlation (Equation (36)) overpredicts the results obtained by present Equation (26) from +3.7% for lower temperature to +14.8% for higher temperature. Contrary to the Hassani et al. correlation, the Khanafer and Vafai correlation (Equation (34)) underpredicts the results obtained by present Equation (26) by ~5.1% within the whole tested temperature range. The values of thermal conductivity for EG-Al_2_O_3_(1 wt%) nanofluid predicted by use of developed Equation (27) are lower by ~3.5% compared to the predictions made by use of the Hassani et al. correlation (Equation (36)) within the whole tested temperature range. The results obtained by the Corcione correlation (Equation (35)) are higher than calculated by present Equation (27), too. The discrepancy increases with temperature increase and amounts 0.8% for lower temperature and 7% for higher temperature. For all three, water-EG/(60:40)Al_2_O_3_(1 wt%), water-EG/(50:50)Al_2_O_3_(1 wt%) and water-EG/(40:60)Al_2_O_3_(1 wt%), nanofluids the literature, correlations overpredict the thermal conductivity within the whole examined temperature range. In the case of the water-EG/(60:40)Al_2_O_3_(1 wt%) nanofluid an excellent agreement between Chiam et al. correlation (Equation (33)) and Hassani et al. correlation (Equation (36)) has been found. The maximum difference between these two correlations does not exceed 1.2%. However, both correlations give higher values of thermal conductivity than present Equation (28), and the discrepancy increases from 5.7% for lower temperature to 12.1% for higher temperature. Sundar et al.’s correlation (Equation (31)) gives higher values of thermal conductivity as well, but the discrepancy is almost constant within the whole temperature range and equals ~8%. In the case of the water-EG/(50:50)Al_2_O_3_(1 wt%) nanofluid, again good agreement between the Chiam et al. correlation (Equation (33)) and Hassani et al. correlation (Equation (36)) has been found. The maximum difference between those two correlations does not exceed 1.7%. However, both correlations give higher values of thermal conductivity than present Equation (29) and the discrepancy increases from 5.4% for lower temperature to 11.7% for higher temperature.

In the case of water-EG/(40:60)Al_2_O_3_(1 wt%) nanofluid, the Chiam et al. correlation (Equation (33)), and Hassani et al. correlation (Equation (36)) give higher values of thermal conductivity than present Equation (30) and the discrepancy increases from 5.2% for lower temperature to 12.1% and 9.3% for higher temperature, respectively. Sundar et al.’s correlation (Equation (32)) gives higher values of thermal conductivity as well, but the discrepancy, in turn, is almost constant within the whole temperature range and equals about 6.2%.

**Figure 9:**
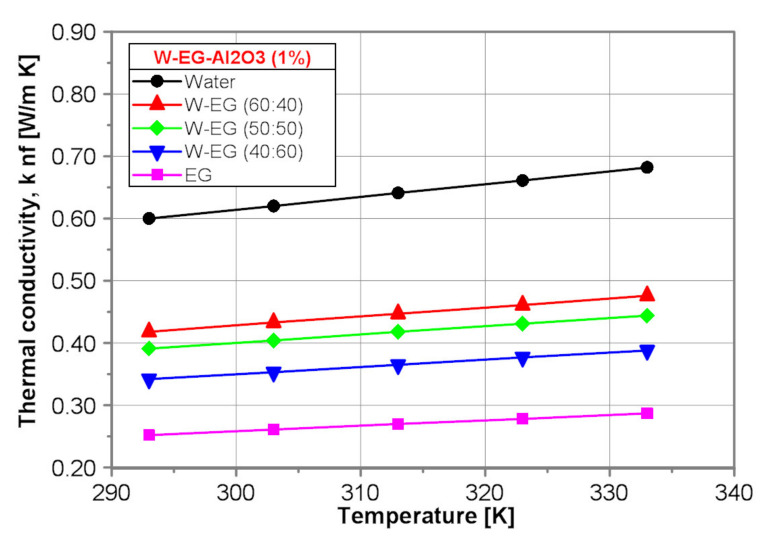
Thermal conductivity of the tested nanofluids.

Figure 9 shows a comparison of the thermal conductivity of the tested nanofluids with nanoparticle mass concentration of 1% obtained by use of the proposed correlation equations presented in Table 5.

Similarly to the base fluids, the thermal conductivity of water-Al_2_O_3_ (1%) nanofluid is the highest one. Moreover, the thermal conductivity of all the tested nanofluids increases with water content and temperature increase.

**Figure 10:**
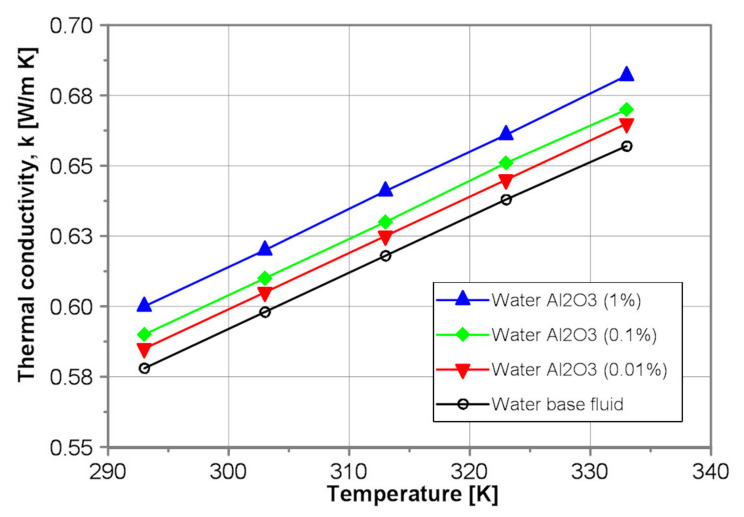
Dynamic viscosity of the tested EG-Al_2_O_3_ nanofluids.

Figure 10, in turn, illustrates exemplarily influence of the nanoparticle concentration on the thermal conductivity of the tested water-Al_2_O_3_ nanofluids. As shown in Figure 10, the addition of alumina nanoparticles results in an increase of thermal conductivity of water-Al_2_O_3_ nanofluids from 1.2% to 3.8% for mass concentration of 0.01% and 1%, respectively.

## 3. Discussion

Both the present developed correlations and some literature correlations, like the correlations of Sundar et al. [15], Chiam et al. [16] and Vajjha and Das [20], used in this paper are purely empirical. This means that these correlations have been developed on the basis of the particular set of experimental data. Therefore, the discrepancy between the calculated values of the dynamic viscosity and thermal conductivity obtained by present correlations and predictions from the mentioned correlations may result for instance from different fabrication methods and nanoparticle dimensions and geometry. It is a well-known fact that fabrication method strongly influences the properties of the nanofluid. For instance—as it was shown in [31]—the sonification time is a decisive factor influencing viscosity and thermal conductivity of the nanofluids.

A major problem for the measurements of thermal conductivity and viscosity is the homogeneity maintenance of the nanofluids. Due to sedimentation and aggregation induced by particle–particle interaction, the nanoparticle concentration and the particle size vary with time changing the thermal conductivity and viscosity of the nanofluids [32,33]. Chen et al. [34] stated that the agglomeration phenomenon could be one of the key parameters responsible for the increase in viscosity. They suggest using the aggregate diameter (d_agg_ = 3.34d_p_) instead of nanoparticle diameter.

Next, there exist no reliable measuring techniques of the thermal conductivity and viscosity of nanofluids. Limitations of the present measuring techniques of thermal conductivity are discussed in [35]. According to [17], the thermal hot wire technique for the thermal conductivity and the stress-controlled rheometer for the dynamic viscosity should be preferred over the KD2pro device and pressure drop/flow rate measurements to get more reliable experimental data. From the other side, as it was shown in [30], application of the rigorous measuring standards may result in distinct reduction of the scatter of experimental data.

As it was discussed in [6,36,37,38,39], more advanced correlations for viscosity and thermal conductivity of nanofluids besides nanoparticle concentration and temperature—as proposed in this paper, should include at least nanoparticle shape and size [6,36,37,38,39]. However, in order to establish the correct trend of variation of the thermal conductivity and viscosity of nanofluids with a number of parameters already mentioned, it is necessary to broad our knowledge about the mechanisms leading to the improvement or deterioration of the thermal conductivity and viscosity of nanofluids as it is recently shown by [40,41]. In the literature several mechanisms potentially responsible for thermal conductivity enhancement of nanofluids are proposed. Commonly accepted mechanism is Brownian motion of nanoparticles [42,43,44]. Formation of nanolayer as an enhancement mechanism has been proposed in [45,46,47]. The enhancement of thermal conductivity of nanofluids due to clustering of nanoparticles has been studied in [48,49]. According to the authors of [50], the dominating thermal conductivity enhancement mechanism of nanofluids is thermophoresis. The ballistic nature of thermal conductivity enhancement of nanofluids has been studied in [48,51]. Other factors responsible for thermal conductivity enhancement of nanofluids are discussed, e.g., in [52,53]. Recently, Mahian et al. [54] comprehensively reviewed the theoretical models of thermal conductivity and viscosity of nanofluids. The readers can refer to other comprehensive reviews such as those in [6,55].

Pure EG is commonly used as a component of antifreeze, coolant and heat transfer fluids in automobiles, aircraft anti-icing/deicing materials, chilled water air conditioning systems and heat transport through geothermal heat pumps [56]. A (50:50) mixture of EG and water is used as the commercial radiator cooler [14]. Other important uses of EG or EG/water mixtures include heat transfer fluids used as industrial coolants for gas compressors, building heating in the sub-arctic and arctic regions, ventilation and ice skating rinks [10,11]. Pure EG is almost an electrical insulator, therefore it can be used as a coolant in electronic systems [9,57]. However, the resistivity of the EG solution decreases with increasing water content. The thermophysical properties of EG/water mixtures vary significantly with based ratio and temperature. It is extremely interesting that the minimum freezing temperature—approximately −55 °C—has a (70:30) mixture of EG and water, while for pure EG it is only −13 °C [58]. Indeed, the content of EG in water solutions determines heat transfer coefficients and as a result cooling capacity and, last but not least, pumping power. As it is shown in the present paper, addition of alumina nanoparticles with mass concentration from 0.01% to 1% has almost no effect on dynamic viscosity of EG-Al_2_O_3_ nanofluids and simultaneously results in thermal conductivity enhancement. This means that there is no increase in pumping power and potential heat transfer augmentation while using EG-Al_2_O_3_ nanofluid as a cooling medium.

## 4. Conclusions

- A comprehensive set of measured thermal conductivity and dynamic viscosity data for pure water, ethylene glycol (EG) and three mixtures of water and EG with volume ratio of 60:40, 50:50 and 40:60 is provided.
- A corresponding set of measured thermal conductivity and dynamic viscosity data for nanofluids with alumina (Al_2_O_3_) nanoparticles dispersed in five base fluids mentioned above is provided as well.
- A set of 20 correlations for the prediction of the thermal conductivity and dynamic viscosity of five base fluids and corresponding nanofluids for mass nanoparticle concentration of 0.01%, 0.1% and 1% and temperature range from 293 K to 333 K has been developed.
- The results reveal that the dynamic viscosity of the base fluids decreases exponentially as the temperature increases, but the dynamic viscosity of EG and water/EG mixtures decreases more rapidly than water.
- It is established that the selected literature correlations for dynamic viscosity match quite well the predictions made by use of present developed correlations for EG and water/EG mixtures based nanofluids. The maximum difference occurs for water based nanofluid with 1% nanoparticle concentration.
- It is established that the thermal conductivity of all tested base fluids and nanofluids is a linear function of temperature.
- The selected literature correlations reproduce reasonably well the thermal conductivity of the tested nanofluids made by use of the proposed present correlations.

## Figures and Tables

**Table 1:** Correlations for the dynamic viscosity of the base fluids.

Liquid	Present Study	Eq.	Vajjha and Das [20]	Equation
Water	$\mu_{bf}=1.435\cdot 10^{-5}\cdot e^{\frac{1227}{T}}$	Equation (1)	-	-
EG	$\mu_{bf}=1.6\cdot 10^{-7}\cdot e^{\frac{3440}{T}}$	Equation (2)	-	-
Water/EG(60:40)	$\mu_{bf}=3.4\cdot 10^{-7}\cdot e^{\frac{2618}{T}}$	Equation (3)	-	-
Water/EG(50:50)	$\mu_{bf}=2.81\cdot 10^{-7}\cdot e^{\frac{2748}{T}}$	Equation (4)	-	-
Water/EG(40:60)	$\mu_{bf}=3.77\cdot 10^{-7}\cdot e^{\frac{2719}{T}}$	Equation (5)	$\mu_{bf}=0.55510^{-3}\;e^{2664/T}$	Equation (6)

**Table 2:** Correlations for the dynamic viscosity of the tested nanofluids.

Liquid	Present Study	Equation
Water	$\mu_{nf}=664.06\varphi_{m}{\;}^{0.0151}t^{0.236}\mu_{bf}^{1.939}$	Equation (8)
EG	$\mu_{nf}=1.11\varphi_{m}{}^{0.0061}\mu_{bf}^{1.017}$	Equation (9)
Water/EG(60:40)	$\mu_{nf}=1.13\varphi_{m}{}^{0.0106}\mu_{bf}^{1.003}$	Equation (10)
Water/EG(50:50)	$\mu_{nf}=1.14\mu_{bf}^{0.9906}$	Equation (11)
Water/EG(40:60)	$\mu_{nf}=2.83\varphi_{m}{}^{0.0094}t^{0.279}\mu_{bf}^{1.3237}$	Equation (12)

**Table 3:** Coefficients A, B and *T*_o_ from Equation (18) for EG-Al_2_O_3_ nanofluids [25].

φ								
	0.000	0.005	0.010	0.015	0.021	0.031	0.048	0.066
A	−3.694	−3.632	−2.381	−1.702	−3.450	−3.302	−1.379	−3.039
B [K]	999.0	999.0	689.3	534.7	999.0	999.0	518.4	999.2
T_o_ [K]	145.7	145.5	169.8	185.5	146.2	145.3	189.9	148.7

**Table 4:** Correlations for the thermal conductivity of the base fluids.

Liquid	Present Study	Eq.	Vajjha and Das [20]	Eq.
Water	$k_{bf}=1.974\cdot 10^{-3}\cdot T$	Equation (19)	-	-
EG	$k_{bf}=8.49\cdot 10^{-4}\cdot T$	Equation (20)	-	
Water/EG(60:40)	$k_{bf}=1.428\cdot 10^{-3}\cdot T$	Equation (21)	-	-
Water/EG(50:50)	$k_{bf}=1.334\cdot 10^{-3}\cdot T$	Equation (22)	-	-
Water/EG(40:60)	$k_{bf}=1.166\cdot 10^{-3}\cdot T$	Equation (23)	$k_{bf}=-0.1057+0.0025\cdot T-3\cdot 10^{-6}T^{2}$	Equation (24)

**Table 5:** Correlations for the thermal conductivity of the tested nanofluids.

Liquid	Present Study	Equation
Water	$k_{nf}=k_{bf}(1+0.1046\varphi_{m}{}^{0.2388}\;{\left(100/d_{p}\right)}^{3.14\cdot 10^{-3}}){\;}_{}$	Equation (26)
EG	$k_{nf}=k_{bf}(1+0.0193{\left(\frac{k_{p}}{k_{bf}}\right)}^{6.15\cdot 10^{-3}}\varphi_{m}{}^{0.0738}\;{\left(100/d_{p}\right)}^{9.76\cdot 10^{-5}}){\;}_{}$	Equation (27)
Water/EG(60:40)	$k_{nf}=k_{bf}$	Equation (28)
Water/EG(50:50)	$k_{nf}=k_{bf}$	Equation (29)
Water/EG(40:60)	$k_{nf}=k_{bf}$	Equation (30)

## Nomenclature

*a*	thermal diffusivity, m^2^/s
*c_p_*	specific heat at constant pressure, J/(kg K)
*d*	diameter, m
*k*	thermal conductivity, W/(m K)
*Pr*	$\mathrm{Prandtl}\;\mathrm{number};\;Pr=\frac{v}{a}=\frac{\mu c_{p}}{k}$
*Re*	Brownian motion Reynolds number; $u_{Br}={\left(\frac{2k_{B}T}{\pi \varrho_{p}d_{p}^{3}}\right)}^{0.5}$
***Greek symbols***	
μ	dynamic viscosity, Pa s
ν	kinematic viscosity, m^2^/s
φ	nanoparticle concentration
ρ	density, kg/m^3^
***Subscripts***	
agg	aggregate
*B*	Boltzmann constant; boiling point temperature
*Br*	Brownian velocity
*bf*	base fluid
*fr*	freezing
m	mass
*nf*	nanofluid
*p*	particle
*v*	volume

## Abbreviations

BR	based ratio
CNT	carbon nanotubes
CPU	central processing unit
EG	ethylene glycol

## References

[1] Taylor R., Coulombe S., Otanicar T., Phelan P., Gunawan A., Lv W., Rosengarten G., Prasher R., Tyagi H. (2013). Small particles, big impacts: A review of the diverse applications of nanofluids. J. Appl. Phys..

[2] Sajid M.U., Ali H.M. (2019). Recent advances in application of nanofluids in heat transfer devices: A critical review. Renew. Sustain. Energy Rev..

[3] Cieśliński J.T. (2019). Application of Nanofluids in Thermal Technologies, Contemporary Issues of Heat and Mass Transfer.

[4] Khanafer K., Vafai K. (2011). A critical synthesis of thermophysical characteristics of nanofluids. Int. J. Heat Mass Transf..

[5] Rashmi W., Khalid M., Ong S.S., Saidur R. (2014). Preparation, thermo-physical properties and heat transfer enhancement of nanofluids. Mater. Res. Express.

[6] Angayarkanni S.A., Philip J. (2015). Review on thermal properties of nanofluids: recent developments. Adv. Colloid Interface Sci..

[7] ASHRAE (2005). ASHRAE Handbook—Fundamentals.

[8] Nazari M., Karami M., Ashouri M. (2014). Comparing the thermal performance water, Ethylene Glycol, Alumina and CNT Nanofluids in CPU Cooling: Experimental study. Exp. Therm. Fluid Sci..

[9] Alfaryjat A., Miron L., Pop H., Apostol V., Stefanescu M.F., Dobrovicescu A. (2019). Experimental Investigation of Thermal and Pressure Performance in Computer Cooling Systems Using Different Types of Nanofluids. Nanomaterials.

[10] Namburu P.K., Das D.K., Tanguturi K.M., Vajjha R.S. (2009). Numerical study of turbulent flow and heat transfer characteristics of nanofluids considering variable properties. Int. J. Therm. Sci..

[11] Sahoo B.C., Vajjha R.S., Ganguli R., Chukwu G.A., Das D.K. (2009). Determination of Rheological Behavior of Aluminum Oxide Nanofluid and Development of New Viscosity Correlations. Petrol. Sci. Technol..

[12] Vajjha R.S., Das D.K. (2009). Specific heat measurement of three nanofluids and development of new correlations. ASME J. Heat Transf..

[13] Vajjha R.S., Das D.K. (2009). Experimental determination of thermal conductivity of three nanofluids and development of new correlations. Int. J. Heat Mass Transf..

[14] Elias M.M., Mahbubul I.M., Saidur R., Sohel M.R., Shahrul I.M., Khaleduzzaman S.S., Sadeghipour S. (2014). Experimental investigation on the thermo-physical properties of Al_2_O_3_ nanoparticles suspended in car radiator coolant. Int. Commun. Heat Mass Transf..

[15] Sundar L.S., Ramana E.V., Singh M.K., Sousa A.C.M. (2014). Thermal conductivity and viscosity of stabilized ethylene glycol and water mixture Al_2_O_3_ nanofluids for heat transfer applications: An experimental study. Int. Commun. Heat Mass Transf..

[16] Chiam H.W., Azmi W.H., Usri N.A., Mamat R., Adam N.M. (2017). Thermal conductivity and viscosity of Al_2_O_3_ nanofluids for different based ratio of water and ethylene glycol mixture. Exp. Therm. Fluid Sci..

[17] Sekrani G., Poncet S. (2018). Ethylene- and Propylene-Glycol Based Nanofluids: A Literature Review on Their Thermophysical Properties and Thermal Performances. Appl. Sci..

[18] Cieśliński J.T., Krygier K., Smoleń S. (2015). Measurement of temperature-dependent viscosity and thermal conductivity of alumina and titania thermal oil nanofluids. Arch. Thermodyn..

[19] Cieśliński J.T., Krygier K., Smoleń S. (2016). Infuence of nanoparticle concentration on thermal properties of thermal oil-MWCNT nanofluids. Appl. Mech. Mater..

[20] Vajjha R.S., Das D.K. (2012). A review and analysis on influence of temperature and concentration of nanofluids on thermophysical properties, heat transfer and pumping power. Int. J. Heat Mass Transf..

[21] Schmidt E. (1969). Properties of Water and Steam in SI-Units.

[22] Ethylene Glycol. Product Guide. https://www.meglobal.biz/wp-content/uploads/2019/10/MEG-0002_MEG_Guide_Rev_Sep_2019.pdf.

[23] Tsierkezos N.G., Molinou I.E. (1998). Thermodynamic Properties of Water + Ethylene Glycol at 283.15, 293.15, 303.15, and 313.15 K. J. Chem. Eng. Data.

[24] Yiamsawas T., Mahian O., Dalkilic A.S., Kaewnai S., Wongwises S. (2013). Experimental studies on the viscosity of TiO_2_ and Al_2_O_3_ nanoparticles suspended in a mixture of ethylene glycol and water for high temperature applications. Appl. Energy.

[25] Pastoriza-Gallego M.J., Lugo L., Legido J.L., Piñeiro M.M. (2011). Thermal conductivity and viscosity measurements of ethylene glycol-based Al_2_O_3_ nanofluids. Nanoscale Res. Lett..

[26] Anoop K.B., Kabelac S., Sundararajan T., Das S.K. (2009). Rheological and flow characteristics of nanofluids: Influence of electroviscous effects and particle agglomeration. J. Appl. Phys..

[27] Patel H.E., Sundararajan T., Das S.K. (2010). An experimental investigation into the thermal conductivity enhancement in oxide and metallic nanofluids. J. Nanopart. Res..

[28] Corcione M. (2011). Empirical correlating equations for predicting the effective thermal conductivity and dynamic viscosity of nanofluids. Energy Convers. Manag..

[29] Hassani S., Saidur R., Mekhilef S., Hepbasli A. (2015). A new correlation for predicting the thermal conductivity of nanofluids; using dimensional analysis. Int. J. Heat Mass Transf..

[30] Buongiorno J., Venerus D.C., Prabhat N., McKrell T., Townsend J., Christianson R., Tolmachev Y.V., Keblinski P., Hu L., Alvarado J.L. (2009). A benchmark study on the thermal conductivity of nanofluids. J. Appl. Phys..

[31] Silambarasan M., Manikandan S., Rajan K.S. (2012). Viscosity and thermal conductivity of dispersions of sub-micron TiO_2_ particles in water prepared by stirred bead milling and ultrasonication. Int. J. Heat Mass Transf..

[32] Ghadimi A., Saidur R., Metselaar H.S.C. (2011). A review of nanofluid stability properties and characterization in stationary conditions. Int. J. Heat Mass Transf..

[33] Lee J., Han H., Koo J. (2014). A novel method to evaluate dispersion stability of nanofluids. Int. J. Heat Mass Transf..

[34] Chen H., Ding Y., He Y., Tan C. (2007). Rheological behaviour of ethylene-glycol-based titania nanofluids. Chem. Phys. Lett..

[35] Paul G., Chopkar M., Manna I., Das P.K. (2010). Techniques for measuring the thermal conductivity of nanofluids: A review. Renew. Sustain. Energy Rev..

[36] Aybar H.Ş., Sharifpur M., Azizian M.R., Mehrabi M., Meyer J.P. (2015). A Review of Thermal Conductivity Models for Nanofluids. Heat Transf. Eng..

[37] Das P.K. (2017). A review based on the effect and mechanism of thermal conductivity of normal nanofluids and hybrid nanofluids. J. Mol. Liq..

[38] Li F., Li L., Zhong G., Zhai Y., Li Z. (2019). Effects of ultrasonic time, size of aggregates and temperature on the stability and viscosity of Cu-ethylene glycol (EG) nanofluids. Int. J. Heat Mass Transf..

[39] Mahbubul I.M., Saidur R., Amalina M.A. (2012). Latest developments on the viscosity of nanofluids. Int. J. Heat Mass Transf..

[40] Selvakumar R.D., Dhinakaran S. (2017). Effective viscosity of nanofluids—A modified Krieger–Dougherty model based on particle size distribution (PSD) analysis. J. Mol. Liq..

[41] Dolatabadi N., Rahmani R., Rahnejat H., Garner C.P. (2019). Thermal conductivity and molecular heat transport of nanofluids. RSC Adv..

[42] Jang S.P., Choi S.U.S. (2004). Role of Brownian Motion in the Enhanced Thermal Conductivity of Nanofluids. Appl. Phys. Lett..

[43] Koo J., Kleinstreuer C. (2005). Impact Analysis of Nanoparticle Motion Mechanisms on the Thermal Conductivity of Nanofluids. Int. Commun. Heat Mass Transf..

[44] Shukla R.K., Dhir V.K. (2008). Effect of Brownian Motion on Thermal Conductivity of Nanofluids. J. Heat Transf..

[45] Yu W., Choi S.U.S. (2003). The Role of Interfacial Layers in the Enhanced Thermal Conductivity of Nanofluids: A Renovated Maxwell Model. J. Nanopart. Res..

[46] Xie H., Fujii M., Zhang X. (2005). Effect of Interfacial Nanolayer on the Effective Thermal Conductivity of Nanoparticle–Fluid Mixture. Int. J. Heat Mass Transf..

[47] Xue Q., Xu W.M. (2005). A Model of Thermal Conductivity of Nanofluids With Interfacial Shells. Mater. Chem. Phys..

[48] Keblinski P., Phillpot S.R., Choi S.U.S., Eastman J.A. (2002). Mechanism of Heat Flow in Suspension of Nano-Sized Particles (Nanofluids). Int. J. Heat Mass Transf..

[49] Wang B.X., Zhou L.P., Peng X.F. (2003). A Fractal Model for Predicting the Effective Thermal Conductivity of Liquid with Suspension of Nanoparticles. Int. J. Heat Mass Transf..

[50] Hwang K.S., Jang S.P., Choi S.U.S. (2009). Flow and Convective Heat Transfer Characteristics of Water-Based Al_2_O_3_ Nanofluids in Fully Developed Laminar Flow Regime. Int. J. Heat Mass Transf..

[51] Avsec J. (2008). The Combined Analysis of Phonon and Electron Heat Transfer Mechanism on Thermal Conductivity for Nanofluids. Int. J. Heat Mass Transf..

[52] Chandrasekar M., Suresh S. (2009). A Review on the Mechanisms of Heat Transport in Nanofluids. Heat Transf. Eng..

[53] Kleinstreuer C., Feng Y. (2011). Experimental and theoretical studies of nanofluid thermal conductivity enhancement: A review. Nanoscale Res. Lett.

[54] Mahian O., Kolsi L., Amani M., Estellé P., Ahmadi G., Kleinstreuer C., Marshall J.S., Siavashi M., Taylor R., Niazmand H. (2019). Recent advances in modeling and simulation of nanofluid flows-Part I: Fundamentals and theory. Phys. Rep..

[55] Udawattha D.S., Narayana M. (2018). Development of a Model for Predicting the Effective Thermal Conductivity of Nanofluids: A Reliable Approach for Nanofluids Containing Spherical Nanoparticles. J. Nanofluids.

[56] Staples C.A., Williams J.B., Craig G.R., Roberts K.M. (2001). Fate, effects and potential environmental risks of ethylene glycol: A review. Chemosphere.

[57] Gayatri M., Sreeramulu D. (2015). Performance of water and diluted ethylene glycol as coolants for electronic cooling. Int. J. Eng. Res. Appl..

[58] Yue H., Zhao Y., Ma X., Gong J. (2012). Ethylene glycol: Properties, synthesis, and applications. Chem. Soc. Rev..

